# The complete chloroplast genome of *Hordeum brevisubulatum*

**DOI:** 10.1080/23802359.2020.1797552

**Published:** 2020-07-25

**Authors:** Xiaoxia Su, Jia Zhao, Zhiyong Wang

**Affiliations:** a Heilongjiang Vocational Institute Ecological, Harbin, P. R. China; b Forest botanical garden of Heilongjiang, Harbin, P. R. China; c Heilongjiang Forestry Design Institute, Harbin, P. R. China

**Keywords:** *Hordeum brevisubulatum*, chloroplast genome, phylogenetic analysis, Gramineae

## Abstract

In this study, the complete chloroplast genome of *Hordeum brevisubulatum* was sequenced and analyzed. The complete chloroplast genome of *Hordeum brevisubulatum* was 137,019 bp in length, encoding a total of 134 genes, including 87 protein-coding genes, 39tRNAs, and 8 rRNAs, with a CG content of 38.27%. The phylogenetic analysis of *Hordeum brevisubulatum* was carried out to determine the position of Gramineae in the phylogenetic evolution.

*Hordeum brevisubulatum* is a perennial herb of the genus *Hordeum* in Gramineae (Sun 1996), usually with rhizome, culm tufted, erect, 40–80 cm high. It is distributed in northeast China, Inner Mongolia, northern Shanxi, Ningxia, Gansu, Qinghai, Xinjiang, Tibet and other provinces and regions (Liu et al. 2009).

*Hordeum brevisubulatum* has strong tillering ability, resistance to poverty and drought, and is a group species of salinized meadow grassland (Tao et al. 2015).

As high yield and good quality herbage, it has good saline-alkali resistance, barren resistance, tramping resistance, cold and drought resistance, etc. (Wang et al. 2013), good production performance and high economic value (Cheng et al. 1996). It is often an ideal herbage variety in the process of building artificial grassland and natural low-wet salinized grassland improvement. *Hordeum brevisubulatum* is also a common forage material used in the quality improvement and breeding of saline-alkali tolerant herbage (Li et al. 2004).

Chloroplast genome as independent units of heredity, mostly unisexual, genetic evolution in the process of restructuring, the choice of pressure is small, can directly reflect the plant in the process of the evolution of the long-term accumulation of genetic variation, and can be used to trace the origin of species and migration, especially the noncoding section of it, because of the rapid evolutionary rate, can distinguish between a variety of haploid type, very suitable for relatives interspecific and intraspecific genetic relationship and genetic diversity research, but also between different species suitable for complex evolution research and phylogenetic status. It is a very effective method to study the genetic structure and evolutionary process of the natural population of species, and has been widely used in plant research.

In this study, the complete chloroplast genome of *Hordeum brevisubulatum* was sequenced and analyzed. Based on the complete chloroplast genome of *Hordeum brevisubulatum* (GenBank MT386010), the Phylogenetic tree was constructed by the maximum likelihood method to determine the phylogenetic position of *Hordeum brevisubulatum*.

The experimental sample was taken from Northeast Forestry University (N45.71841°, E126.63178°), the voucher specimen (NEFI20200501wang01) was deposited at Herbarium, Northeast Forestry University, Harbin, China. University.

Chloroplast genome mapping was made by using OGDRAW (https://chlorobox.mpmpg.golm.mpg.de/ogdraw.html). Using online tools IRscope (https://irscope.shinyapps.io/irapp/) to complete the IR expansion shrinkage of the region and the boundary analysis. RAxML software was used to construct the evolutionary tree of chloroplast genome (Figure 1).

**Figure 1.**
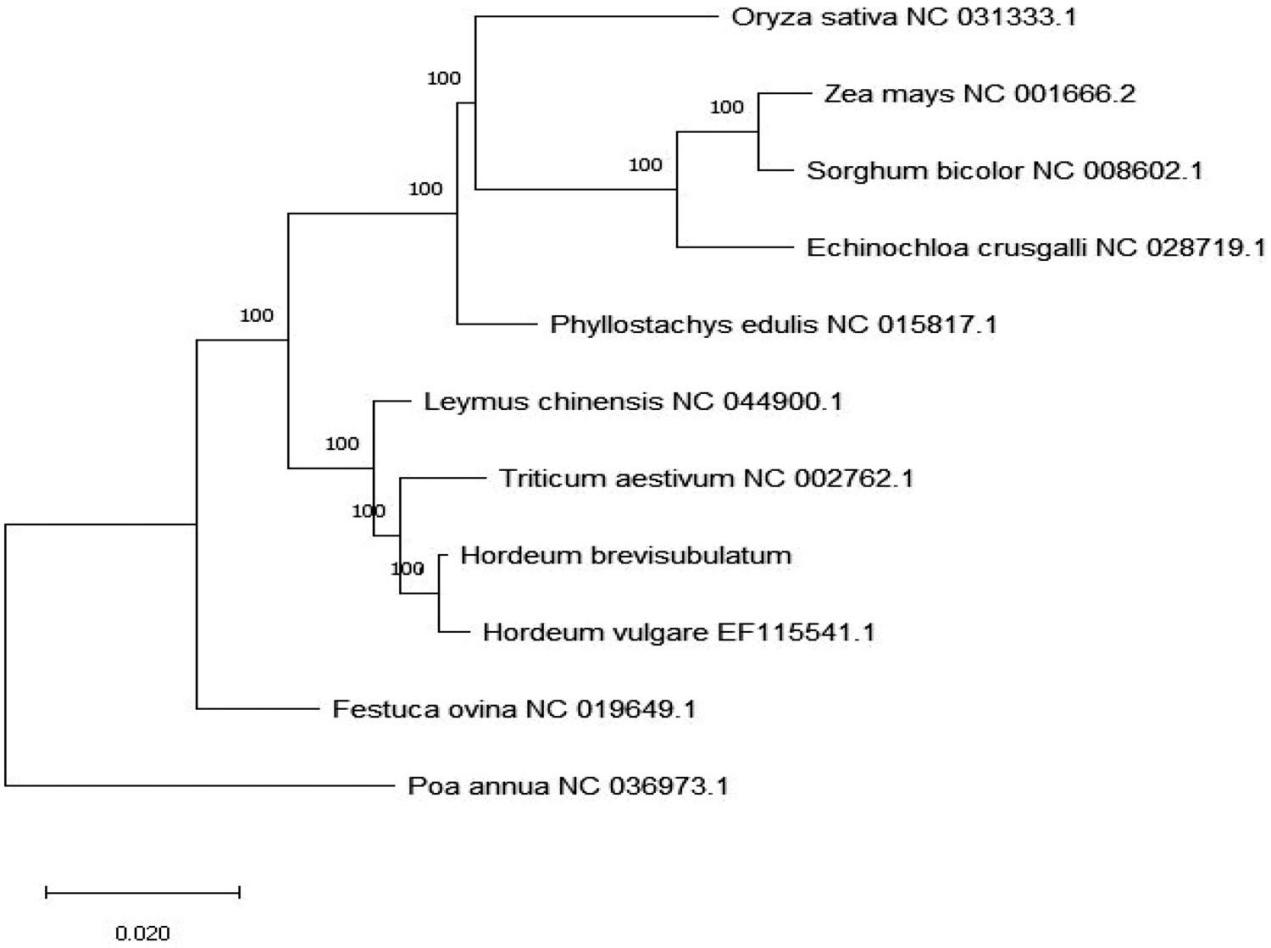
Phylogenetic tree inferred by Maximum Likelihood (ML) method based on the complete chloroplast genome of 10 species of Gramineae and taking Poa annua as an outgroup.

The chloroplast genome of *Hordeum brevisubulatum* was 137019 bp long and showed a typical four-stage structure, including a large single-copy area (LSC) with a length of 81141 bp, and a small single-copy area (SSC) with a length of 12732 bp, and two reverse repeat area (IRA/IRB), both of which were 21573 bp.The content of CG in whole chloroplast genome was 38.27%, including 36.24% in LSC region, 32.25% in SSC region and 43.87% in IR region. A total of 134 genes were encoded, including 87 protein-coding genes, 39 tRNA genes and 8 rrna genes. Among them trnk-uuu, rps16, atpF, ycf3, trnl-uaa, trnv-uac, rps12, petB, petD, rpl16, rpl2, ndhB, rps12, trni-gau, trna-ugc, ndhA, trna-ugc, trni-gau, ndhB, rpl2 contain a single intron, and two introns are contained in ycf3, rps12 and rps12.Using the complete chloroplast genome of *Hordeum brevisubulatum* and the complete chloroplast genome information of ten species of Gramineae plants obtained from NCBI, the Phylogenetic tree was constructed by the maximum likelihood method. Phylogenetic analysis showed that *Hordeum brevisubulatum* is the closest relative to *Hordeum vulgare*, and is in the same branch as *leymus chinensis* and *Triticum aestivum*.

## Data Availability

The *Hordeum brevisubulatum* data has been stored in nucleotide database of National Center of Biotechnology Information. GenBank accession number is MT386010. All the information can be found on the website (https://www.ncbi.nlm.nih.gov/nuccore/MT386010).
